# Histochemical and Microscopic Studies Predict that Grapevine Genotype “Ju mei gui” is Highly Resistant against *Botrytis cinerea*

**DOI:** 10.3390/pathogens9040253

**Published:** 2020-03-31

**Authors:** Mati Ur Rahman, Qingqing Ma, Bilal Ahmad, Muhammad Hanif, Youlin Zhang

**Affiliations:** 1 College of Food Engineering and Nutritional Science, Shaanxi Normal University, Xi’an 710119, China; mati@snnu.edu.cn; 2 Ecological Agricultural Station, Weinan Agricultural Technology Promotion Center, Weinan 714000, Shaanxi, China; maq1745@gmail.com; 3 College of Horticulture, Northwest A&F University, Yangling 712100, China; bajwa1999@nwafu.edu.cn; 4 Horticultural Research Institute, National Agricultural Research Center, Islamabad 45500, Pakistan; mhanif@nwafu.edu.cn

**Keywords:** *Botrytis cinerea*, reactive oxygen species, jasmonic acid, antioxidant enzymes, scanning electron microscope, physiology, “Summer black”, “Ju mei gui”

## Abstract

The necrotrophic fungus *Botrytis cinerea* causes devastating pre- and post-harvest yield losses in grapevine (*Vitis vinifera* L.). Although *B. cinerea* has been well-studied in different plant species, there is limited information related to the resistance and susceptibility mechanisms of *Vitis* genotypes against *B. cinerea* infection. In the present study, leaves and berries of twenty four grape genotypes were evaluated against *B. cinerea* infection. According to the results, one genotype (Ju mei gui) was highly resistant (HR), one genotype (Kyoho) was resistant (R), eight genotypes were susceptible (S), and fourteen genotypes were highly susceptible (HS) against infection of *B. cinerea* in leaves. Whereas in the case of *B.* *cinerea* infection in grape berry, three genotypes were found to be highly resistant, three resistant, eleven genotypes susceptible, and seven were highly susceptible. To further explore the mechanism of disease resistance in grapevine, we evaluated “Ju mei gui” and “Summer black” in terms of *B. cinerea* progression, reactive oxygen species reactions, jasmonic acid contents, and the activities of antioxidant enzymes in leaf and fruit. We surmise that the resistance of “Ju mei gui” is due to seized fungal growth, minor reactive oxygen species (ROS) production, elevated antioxidant enzyme activity, and more jasmonic acid (JA) contents. This study provides insights into the resistance and susceptibility mechanism of *Vitis* genotypes against *B. cinerea*. This will help for the selection of appropriate germplasm to explore the molecular basis of disease resistance mechanisms in grapevine.

## 1. Introduction

Grape (*Vitis vinifera* L.) is an extensively cultivated crop that has vast economic importance as it is a source of numerous products [1], though the berry quality and yield of grapevine is restricted by many abiotic and biotic stresses [2]. *B. cinerea* is a necrotrophic fungus that causes overwhelming grey mold disease. This pathogen is the second most widespread plant pathogen accountable for pre- and post-harvest dwindling and fruit quality worsening [3]. This necrotrophic fungus actively attempts to destroy the living host tissues and naturally senesced plant tissues to use them as nutrients [4] where periods of cold temperatures (18–22 °C) and relative humidity (more than 90%) persist for a long time [5]. The pathogen causes reduction both in quality and yield of wine [6]. Host disease development depends on various traits, such as bunch compactness, morphological, anatomical, and chemical features of the berry skin, which are highly reliant on the grapevine cultivar [7]. 

The traditional control of *B. cinerea* includes strong fungicide treatments during the seasonal crop cycle, but the excessive use of fungicides has many negative effects including increase in production cost, development of fungicide resistant strains, and environmental pollution [8,9]. Thus, the development of disease resistant cultivars is the dire need of time. Most cultivated species of *V. vinifera* are susceptible to many diseases, and the susceptibility differs among the cultivars [10]. In this experiment, the disease signs and symptoms were assessed in a total of 24 grape genotypes at various stages of grape and *B. cinerea* interactions. Additionally, the contents of reactive oxygen species were calculated, which play important functions in plant physiology, comprising development, cellular signaling, and biotic and abiotic stress tolerance. Reactive oxygen species (ROS) production must be firmly controlled to stabilize the biological functions [11]. Considerable confirmation shows that *B. cinerea* challenges can initiate the ROS stress on plants [12]. 

Redox reactions regulate numerous cellular signaling activities and may be directly involved in the cellular redox metabolism [13]. The plant and fungus association is related with ROS production. Oxidative rupture is an initial and universal plant response to pathogen attack. In *B. cinerea*, plant cell death is favorable to the pathogen and causes susceptibility of the host [14]. Antioxidants avoid and shield the cell from the damage caused by free radicals, which help in sustaining the rate of oxidation reactions in a cell [15] and play a very critical role in mitigating the process of oxidation of other molecules [16]. To avoid the oxidative damage caused by these toxic ROS, the level of the endogenous antioxidant defense system is raised in higher plants [17]. Furthermore, Jasmonic acid (JA) is known to be involved in biotic stress amelioration in plants [18] and plays a key governing function in defense responses to necrotrophs [19], also contributing to reactions to insect and pathogen attacks [20]. Plant hormones like jasmonic acid are involved in biotic stress neutralization [18]. JA plays an important role in the stimulation of induced systemic resistance in plants to pathogen or pest attack and accumulates rapidly in plant tissues after exposure to fungal elicitors [21]. It has also been reported that JA and its methyl ester (MeJA) is involved in plant defense mechanisms against biotic and abiotic stresses [22]. 

The main objective of this work was to evaluate different grapevine genotypes against *B. cinerea* by using grape leaves and berries under controlled conditions. Furthermore, we explored the ROS contents, antioxidant enzymes and JA contents in “Ju mei gui” highly resistant (HR) and “Summer black” highly susceptible (HS) genotypes. This study provides information regarding resistance and susceptibility mechanisms of *Vitis* genotypes that may assist in future breeding programs. 

## 2. Results

### 2.1. Grape Genotypes and Their Various Levels of Resistance to B. cinerea

Twenty-four grape genotypes were evaluated to investigate the resistance level of leaves against *B. cinerea.* One genotype was classified as HR, one as resistant, eight as susceptible (S), and fourteen as HS (Table 1). Similarly, *Vitis* genotypes were evaluated for berry resistance level against *B. cinerea* infection, three genotypes were HR, three resistant (R), eleven genotypes S, and seven were HS (Table 2). Grapevine genotypes revealed various grades of resistance to *B. cinerea*. Leaf and berry observations were used to assess *B. cinerea* infection [23] and the range of leaf and berry lesions caused by *B. cinerea* were quantified at 72 hpi (hours post inoculation) (Table 1) and 8 dpi (days post inoculation), respectively (Table 2). Few grape genotypes showed substantial variations in *B. cinerea* resistance (Table 1 and Table 2), and a least significant difference (LSD) test showed resemblance among the replicates, and average disease severity was considerably different (*p* > 0.05) among the various genotypes (Table 1 and Table 2). 

**Figure 1:**
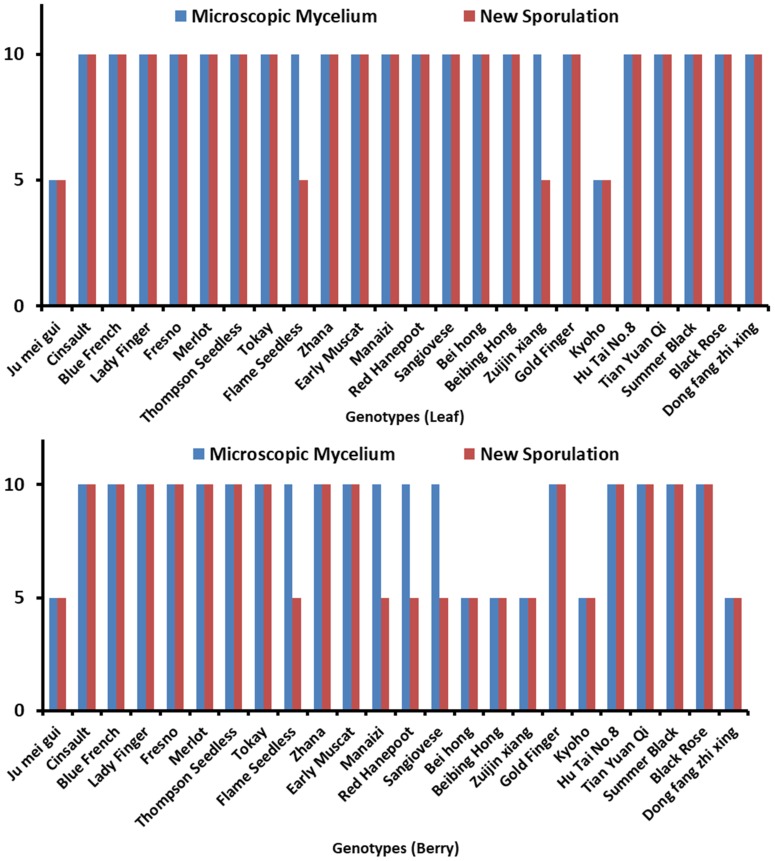
Demonstrating different *Vitis* genotypes. Microscopic mycelium and new sporulation in (**A**) leaves and (**B**) berries, whereas 5 = absence of microscopic mycelium and new sporulation, while 10 = presence of microscopic mycelium and new sporulation.

Microscopic mycelium and new sporulation was also observed in inoculated leaves and berries of various genotypes as shown in Figure 1. The leaves and berries of 24 genotypes were assessed to reveal the resistance level against *B. cinerea*. According to observations, leaves and berries were categorized according to their disease severity index (SI) at 72 hpi and 8 dpi, respectively. Among 24 genotypes, 14 (Table 1) were HS in leaves evaluation while 7 (Table 2) were revealed in berries according to a disease SI of 5.51–7.0. Microscopic mycelium and new sporulation was witnessed on these genotypes. A total of 8 genotypes (Table 1) were S in leaves and 11 (Table 2) in berries evaluation, with mycelium production at 72 hpi, with less/no sporulation (SI of 3.51–5.50). 

### 2.2. Fungal Growth on Leaves and Berries Post B. cinerea Inoculation

**Figure 2:**
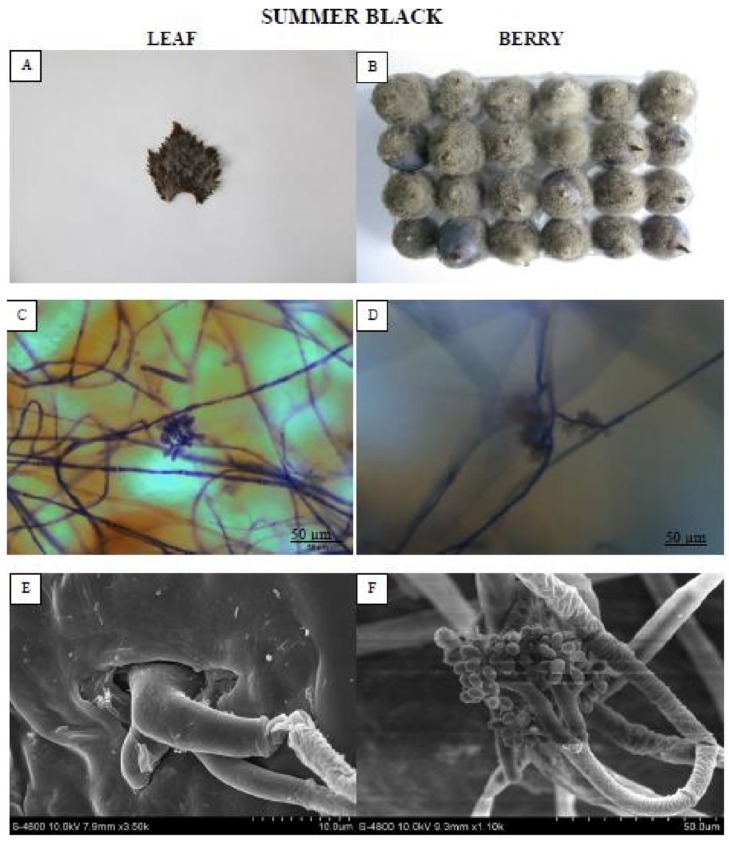
Showing the highly susceptible (HS) “Summer black” leaf and berry post *B. cinerea* inoculation comparison by phenotypic (**A**,**B**), microscopic (**C**,**D**) and electron microscopic (**E**,**F**) study. Scale bars (**C**,**D**): 50 µm; (**E**,**F**): 20 µm. Samples were collected 72 hpi and 8 dpi, respectively.

One representative genotype each from the HR and HS categories was selected for macroscopic, microscopic, and SEM evaluation of fungal colonization on leaves and berries at 72 hpi and 8 dpi, respectively. The leaf of “Summer black” (Figure 2) genotype was entirely enclosed in mold and was roofed by mycelium as well as new sporulation. 

**Figure 3:**
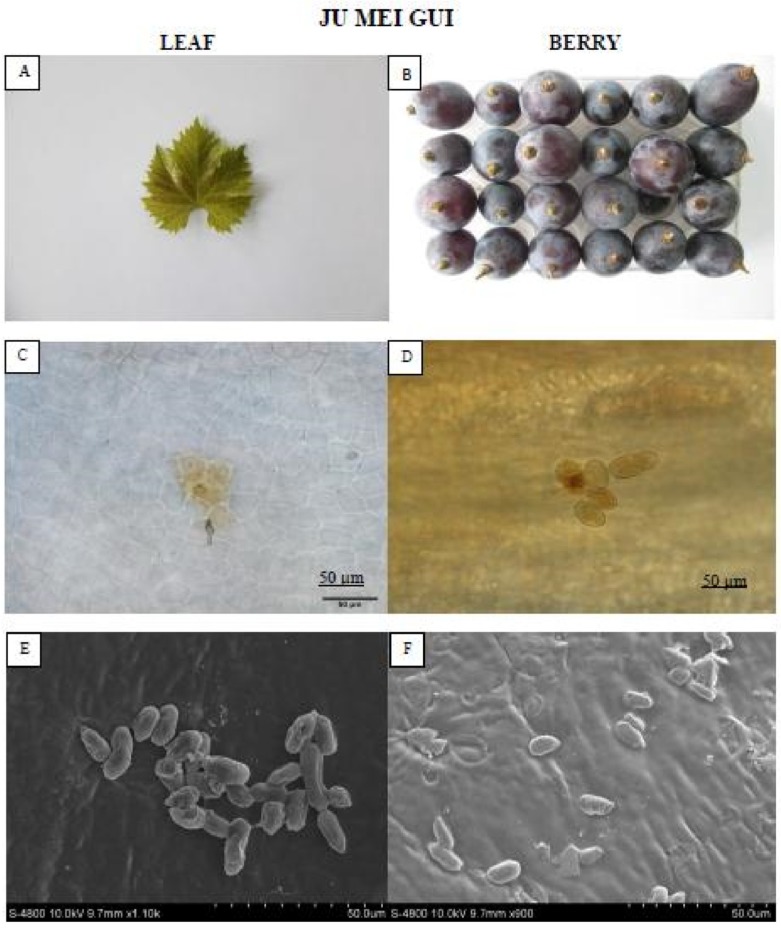
Showing the HR “Ju mei gui” leaf and berry post *B. cinerea* inoculation comparison by phenotypic (**A**,**B**), microscopic (**C**,**D**) and electron microscopic (**E**,**F**) study. Scale bars (**C**,**D**): 50 µm; (**E**,**F**): 20 µm. Samples were collected 72 hpi and 8 dpi respectively.

The “Ju mei gui” (Figure 3) formed no necrotic lesions compared to “Summer black.” Moreover, conidia were observed on the leaves and berries of “Ju mei gui” though the subsequent hyphae was absent, representing restrained *B. cinerea* growth. 

### 2.3. Peroxidase and Superoxide Dismutase Activities in ”Ju mei gui” and “Summer black” Post B. cinerea Inoculation

#### 2.3.1. Activities of Superoxide Dismutase (SOD)

**Figure 4:**
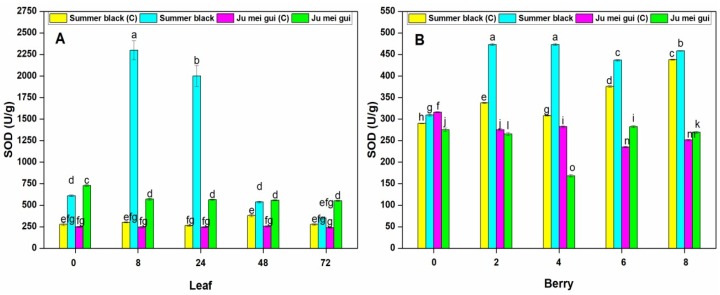
Superoxide dismutase (SOD) activities of “Ju mei gui” and “Summer black” leaves (**A**) and berries (**B**) at 0, 8, 24, 48, 72 h post-inoculation (hpi) and 0, 2, 4, 6, 8 days post-inoculation (dpi), respectively, with Botrytis suspension and using sterile water as the control. The letter “C” with variety name stands for control. Small letters indicate significant differences according to an LSD test (*p* < 0.05) between “Ju mei gui” and “Summer black.”

Superoxide dismutase (SOD) and peroxidase (POD) were measured in the infected and control leaves and berries. Stress circumstances interrupt ROS production resulting in plant cell decease, and plants make an arrangement of antioxidant enzymes to hunt destructive ROS and defend cells from oxidative injury [24]. The SOD activities were determined in the infected and control leaves (Figure 4A) of HR “Ju mei gui”and HS “Summer black.” The SOD activities in “Ju mei gui”and “Summer black” inoculated and control was approximately the same at all-time points (0, 8, 24, 48, 72 hpi). However, the highest peak was detected at 8 hpi followed by 24 hpi in “Summer black” inoculated.

Similarly, we observed the SOD activities at different time points (0, 2, 4, 6, 8 dpi) in berries (Figure 4B) for “Ju mei gui”and “Summer black.” Elevated levels were observed in “Summer black” inoculated, followed by “Summer black” control at 2 dpi, followed by 4 dpi and prolonged until 8 dpi. According to our observations, SOD levels were higher in inoculated leaves (Figure 2A) as related to inoculated berries (Figure 2B) at 8 hpi and 2 dpi, respectively. 

#### 2.3.2. Activities of Peroxidase (POD)

**Figure 5:**
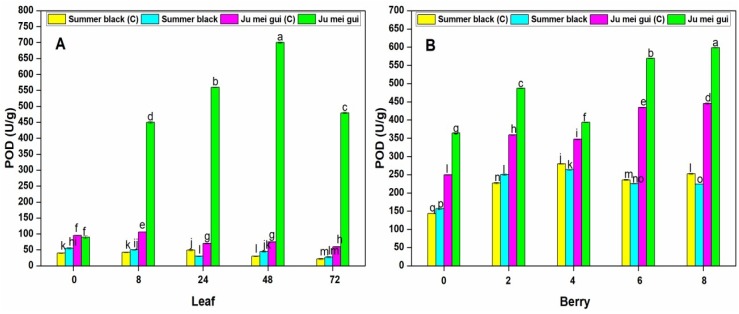
Peroxidase (POD) activities of “Ju mei gui” and “Summer black” leaves (**A**) and berries (**B**) at 0, 8, 24, 48, 72 h post-inoculation (hpi) and 0, 2, 4, 6, 8 days post-inoculation (dpi), respectively, with Botrytis suspension and using sterile water as the control. The letter “C” with variety name stands for control. Small letters indicate significant differences according to an LSD test (p < 0.05) between “Ju mei gui” and “Summer black.”

The POD activities were observed in the infected and control leaves (Figure 5A) at various time points (0, 8, 24, 48, 72 hpi) of “Ju mei gui” and “Summer black.” The POD activities in the “Ju mei gui”and “Summer black” inoculated and control were approximately the same at 0 hpi. However, a gradual increase was observed in “Ju mei gui” inoculated at 8 hpi, followed by 24 hpi and 48 hpi, and then slightly decreased at 72 hpi. Similarly, we observed the SOD activities in berries (Figure 5B) for the HR and HS genotypes, and the elevated levels were observed in “Ju mei gui” inoculated, followed by “Ju mei gui” control at all-time points (0, 2, 4, 6, 8 dpi). By comparing the leaves and berries POD activities, POD levels were higher in inoculated leaves (Figure 3A) compared to inoculated berries (Figure 3B) at 8 hpi and 2 dpi, respectively.

### 2.4. Hydrogen Peroxide (H_2_O_2_) Accumulation in HR ”Ju mei gui” and HS “Summer black”leaves and berries in Response to Infection with B. cinerea

#### 2.4.1. H_2_O_2_ Activities in “Ju mei gui” and “Summer black” Following Inoculation with *B. cinerea*

**Figure 6:**
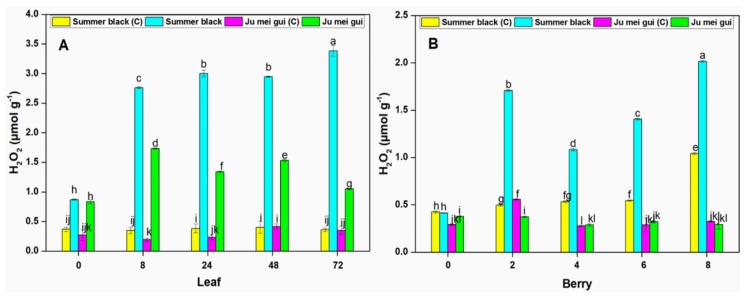
Hydrogen peroxide (H_2_O_2_) contents of “Ju mei gui” and “Summer black” leaves (**A**) and berries (**B**) at 0, 8, 24, 48, 72 h post-inoculation (hpi) and 0, 2, 4, 6, 8 days post-inoculation (dpi), respectively, with Botrytis suspension and using sterile water as the control. The letter “C” with variety name stands for control. Small letters indicate significant differences according to an LSD test (*p* < 0.05) between “Ju mei gui” and “Summer black.”

The H_2_O_2_ activities were measured at various time points (0, 8, 24, 48, 72 hpi) in the infected and control leaves (Figure 6A) of HR “Ju mei gui”and HS “Summer black.” The H_2_O_2_ activies in the “Ju mei gui”and “Summer black” inoculated and control were approximately the same at 0 hpi. However, a gradual elevation was observed at 8 to 72 hpi followed by “‘Ju mei gui” inoculated (Figure 6A). Likewise, we observed the H_2_O_2_ activities at different time points (0, 2, 4, 6, 8 dpi) in berries (Figure 6B) for the “Ju mei gui”and “Summer black” and found that there was no difference between all treatments at 0 hpi while a sudden increase was observed at 2 dpi and dropdown at 4 dpi but again a gradual increase was observed at 6 dpi followed by 8 dpi (Figure 6B).

H_2_O_2_ levels were higher in inoculated leaves (Figure 6A) compared to inoculated berries (Figure 6B) at various time points.

#### 2.4.2. Superoxide Radicals (O2-) Activities in “Ju mei gui” and “Summer black” Post-*B. cinerea* Inoculation

**Figure 7:**
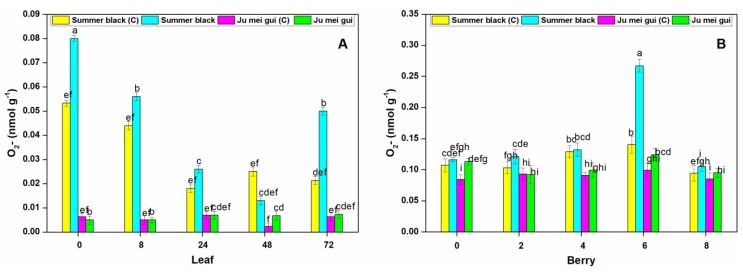
Superoxide radical (O2-) contents of “Ju mei gui” and “Summer black” leaves (**A**) and berries (**B**) at 0, 8, 24, 48, 72 h post-inoculation (hpi) and 0, 2, 4, 6, 8 days post-inoculation (dpi), respectively, with Botrytis suspension and using sterile water as the control. The letter “C”with variety name stands for control. Small letters indicate significant differences according to an LSD test (*p* < 0.05) between “Ju mei gui”and “Summer black.”

The O2- activities were measured at various time points (0, 8, 24, 48, 72 hpi) in inoculated and control leaves (Figure 7A) of HR “Ju mei gui” and HS “Summer black”. The O2- activities in the “Summer black” inoculated and control were highest at 0 hpi, followed by 8 hpi. However, a sudden decrease was observed at the rest of time points (Figure 7A). We also observed the O2- activities at different time points (0, 2, 4, 6, 8 dpi) in berries (Figure 7B) for the HR and HS genotypes and no significant differences were found at various time points, except a sudden increase observed in “Summer black” inoculated at 6 dpi (Figure 7B). 

### 2.5. Jasmonic Acid Contents in Leaves and Berries of “Ju mei gui” and “Summer black” with Post-B. cinerea Inoculation

**Figure 8:**
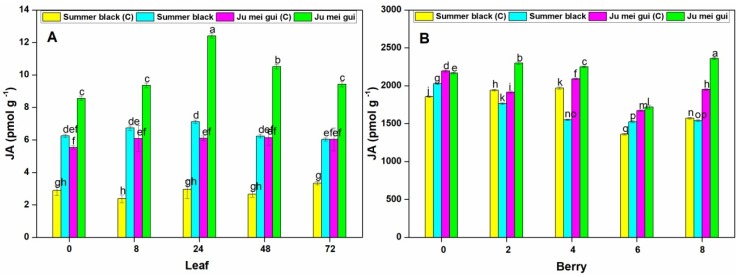
Jasmonic acid (JA) activities of protein extracts from “Ju mei gui” and “Summer black” leaves (**A**) and berries (**B**) at 0, 8, 24, 48, 72 h post-inoculation (hpi) and 0, 2, 4, 6, 8 days post-inoculation (dpi), respectively, with Botrytis suspension and using sterile water as the control. The letter “C” with variety name stands for control. Three independent experiments were used for the means and standard errors. Small letters indicate significant differences according to an LSD test (*p* < 0.05) between “Ju mei gui” and “Summer black.”

The JA activities were measured at various time points (0, 8, 24, 48, 72 hpi) in the inoculated and control leaves (Figure 8A) of “Ju mei gui” and “Summer black.” The JA activities in the “Ju mei gui” and “Summer black” inoculated were higher than that of control treatments. An increase was observed at all-time points with the highest peak at 24 hpi in “Ju mei gui” followed by “Summer black” inoculated and “Ju mei gui”control (Figure 8A). Moreover, the “Summer black” inoculated and “Ju mei gui”control were approximately the same at all-time points. Likewise, we observed the JA activities at different time points (0, 2, 4, 6, 8 dpi) in berries (Figure 8B) for “Ju mei gui”and “Summer black”, and found that there was no difference between almost all treatments, except for higher levels of “Ju mei gui”inoculated at all-time points (Figure 8B).

## 3. Discussion

*B. cinerea* is particularly challenging as it not only devastates green tissue, decreasing yield potential, but also can infect fruit [25]. The world’s population is predicted to rise to more than 9.7 billion by 2050, and worldwide crop production should be doubled in order to meet the increased demand for food. Reducing yield losses to pests and diseases will be a key step in the direction of achieving this challenge [26]. Thus, in light of previous studies, it is necessary to protect crops from pests and diseases to ensure food safety and to develop disease resistant cultivars to reduce the problem of yield and quality losses. In this study, grape genotypes were evaluated against *B. cinerea* infection by using grape leaves and berries. *Vitis* genotypes differ in expressions of their resistance to infection, score of fungal growth and disease severity to *B. cinerea* [27]. Here, 24 different *Vitis* genotypes were evaluated for disease resistance in leaves and berries. According to the results, cultivars were divided into highly resistant, resistant, susceptible, and highly susceptible (Table 1 and Table 2). 

Distinct growth of *B. cinerea* on grape leaves (72 hpi) and berries (8 dpi) was studied phenotypically, microscopically, and via scanning electron microscopy (SEM). In “Summer black,” the pathogen infection feasted significantly and showed signs of sporulation on 72 hpi, whereas in “Ju mei gui” leaves, fungal growth was considerably stuck as revealed by the lesser germination and infection rates. On the “Ju mei gui” leaves, maximum *B. cinerea* conidia failed to develop into infection pegs, which are in line with previous reports [28]. Similarly, sporulation masses on Langao-5 (*V. davidii*) and Baihe-35-1 (*V. pseudoreticulata*) were considerably lesser as compared to the HS cultivar Pinot noir (*V. vinifera*) [11]. ROS production is conjoint in response to pathogen occurrence and elevated contents of ROS were detected in “Summer black.” This is in accordance with previous studies where host–pathogen interactions ROS accretions endorse pathogen growth, disease development, and assist colonization on leaves. In genotype “Ju mei gui”, little amounts of ROS post-inoculation were detected, signifying that the antioxidant enzymes uphold redox equilibrium and shield cells from ROS destruction [17,26]. 

Oxidative stress interrupts the redox equilibrium in diseased tissues, thereby facilitating disease progression [29]. In this study, elevated ROS accumulation after inoculation was noticed in leaves and berries of “Summer black.” We determined that “Summer black” was extensively affected from the constant presence of ROS, and that “Ju mei gui” was not capable of experiencing significant oxidative stress because of a timely initiated antioxidative system. H_2_O_2_ production is induced in plant cells and accompanied by O2- generation, which can encourage programmed cell death and disease lesion expansion, thus increasing *B. cinerea* infection [30]. H_2_O_2_ in higher or lower contents increase either the susceptibility or resistance, respectively, to *B. cinerea*, while O2- serves as a first substrate for H_2_O_2_ formation [14,29,31] and supports *B. cinerea* attack [32]. “Summer black” leaves showed minor differences in POD activity with lesion development post-inoculation. Conversely, they showed elevated SOD activity, which is related with H_2_O_2_ production and O2- decline. The increased levels of POD activity were found in “Ju mei gui” and no substantial alteration was detected in SOD activity. Small amounts of ROS production are necessary for the antioxidative system to maintain redox equilibrium [33], and we also detected that the infected “Summer black” exhibited an inadequate antioxidative system, causing constantly higher ROS production. 

Previously, it has been reported that the resistant genotype “Pingli-5” produced low ROS production and activated its antioxidant enzymes when interacting with *B. cinerea,* which is correlated with its pathogen resistance. Meanwhile, susceptible Red globe experienced severe pathogen infection and continuously produced ROS and was found to have relatively inactive antioxidative responses [28]. On the other hand, “Ju mei gui” displayed comparatively rapid changes in antioxidative capacity, particularly POD activity, and less ROS-induced stress. Significantly higher levels of ROS were detected in “Summer black” but not in “Ju mei gui” which may be a main feature in the capability of genotype “Ju mei gui” to protect itself against *B. cinerea*. These findings are in line with the previous studies in that the coordination between ROS production and scavenging mechanisms are related to the antioxidative system during biotic stress [34]. 

JA contents were measured in both leaves and berries of “Ju mei gui” and “Summer black” genotypes, and higher contents were found in “Ju mei gui.” We noted high JA contents in the “Ju mei gui” control, which were nearly equivalent to the JA contents observed in inoculated “Summer black” (Figure 8), which suggests that the presence of more contents of JA in “Ju mei gui” may act to control *B. cinerea*. These results are in accordance with the studies reported that high JA contents block *B. cinerea* infection and reinforce grape resistance to *B. cinerea* [35]. Furthermore, JA is a vital component in the plant defense responses against insects and microbial pathogens [36], is a major hormone involved in plant defense responses [37] and its production occurs relatively rapidly in plant tissues after interactions with fungal elicitors [21,38].

Finally, the resistance of “Ju mei gui” can attribute to minor fungal growth, less ROS production, elevated antioxidant enzyme activities, and more JA contents. Furthermore, serious fungal infection of “Summer black” and persistent ROS detection coincide with rather unaffected antioxidant functions and minute JA contents. This study provides an understanding into *B. cinerea* infection in grapevine, which can be a precious source for researchers by providing information for choosing appropriate grapevine genotypes.

## 4. Materials and Methods

### 4.1. Grape and Fungal Resources

Grape (leaves & berries) were acquired from the Grapevine garden (340 12’N, 1080 07’E) of Northwest Agriculture and Forestry University, Yangling, Shaanxi, China. The area is located 520 m above sea level. Annual mean temperature and rainfall are 12.9 °C and 660 mm, respectively. Maximum rainfall occurs between July and September. Twenty plants for each genotype were used for leaf and berry assessments. Leaves and berries were collected on the dates in the year 2019, i.e., June 5, (genotypes 1 to 10), June 11, (genotypes 11 to 20), and June 16, (genotypes 21 to 24). Before starting the experiment, *B. cinerea* spores were isolated from “Flame seedless” cultivar (*V. vinifera*) grown in a greenhouse located on the North campus of the Northwest A&F University, Shaanxi, China. Spores were cultured on a potato dextrose agar (PDA) medium at 22 °C. *B. cinerea* developed white to gray colonies on potato dextrose agar (PDA) culture medium and produced filamentous, hyaline, branched and septate mycelia with prominent cell walls. The conidia were unicellular, hyaline to slightly colored, smooth, ovoid to ellipsoid. Furthermore, the conidia were produced on short sterigmata on the swollen tips of aerial, branched conidiophores. Black, melanized, elongated or spherical sclerotia were produced under unfavorable conditions in vitro, which play an important role in pathogen survival, dispersal and multiplication. As far as the growth of the pathogen is concerned, the following developments were observed: at 4 hpi, there was no significant change; at 8 hpi, appressoria development was initiated and became visible at 12 hpi under microscope. The multiplication rate increased after 18 hpi with development of infection pegs; the hyphae were developed after 36 hpi and mycelium growth was seen after 48 hpi; the mycelium was increased on the subsequent time points till the sporulation; sporulation started at 120 hpi and covered the whole surface on day 21 having new sporulation. After this, the conidia were removed, and an optimum concentration of 1.5 x 106 spores·mL^−1^ for leaf assessment was prepared in sterilized water as earlier described by [28,39]. Similarly, for berry evaluation an optimum concentration of 1 × 105 spores·mL^−1^ was prepared [39,40]. The conidia suspension was assured to have a conidia/spore germination percentage of 95% or more before all experimentations.

### 4.2. Detached Leaf and Berry Evaluation

Detached leaf evaluation post-inoculation was done according to previous methods [28,39]. Leaves of the same size and age (from the shoot at nodes 3 and 4) were arbitrarily selected from the grape plants. The detached leaves were washed with distilled water. For laboratory assessment, 48 leaves from each of three replicates of each genotype were evaluated. The leaves were quickly transferred to trays with 0.8% agar and sprayed evenly with the conidial suspension. Control leaves were sprayed with distilled water. The trays were placed in an incubator with a relative humidity of 90–100%, for the first 24 h in the dark and then 12/12 h light/dark at 22 °C.

Berry evaluation was performed using grape berries of the same size and age, E-L 39 stage, as it is the optimum stage to study *B. cinerea* disease reactions [39,40,41]. Berries were randomly selected and harvested from the grape plants and washed several times with distilled water for research evaluation. One hundred and five berries from three replicates of individual genotypes were assessed. The berries were sprayed uniformly with the conidial suspension. Control berries were sprayed with distilled water and kept in an incubator with a relative humidity of more than 90% at 22 °C for 8 days duration.

### 4.3. Disease Severity Assessment

Disease severity was evaluated and rated as previously described [42] with slight modifications. The disease symptoms observed on the leaves were ranked from 1 to 7 (Rank 1 = 0.1–5.0%, 2 = 5.1–15.0%, 3 = 15.1–30.0%, 4 = 30.1–45.0%, 5 = 45.1–65%, 6 = 65.1–85.0% and 7 = 85.1–100.0%) on the basis of the estimated percentage of lesions over the entire leaf and berry surface. The ranking was then converted into a severity index (SI) according to the formula:

(1) $$\mathrm{SI}=\frac{\sum \left(Rank\;x\;number\;of\;infected\;leaves/berries\;in\;that\;rank\right)}{Total\;number\;of\;leaves/berries\;x\;highest\;rank}\;\times \;100$$

The resistance level was rated into four classes on the basis of the SI values. Disease resistance levels of the different genotypes were categorized as highly resistant (SI: 0–1.50), resistant (SI: 1.51–3.50), susceptible (SI: 3.51–5.50), and highly susceptible (SI: 5.51–7.0). Susceptibility data for the disease were collected in 2019. The average SI values of the data were used to evaluate the resistance level.

### 4.4. Microscopic Assessment of B. cinerea Development

One representative genotype from each category was used to characterize the colonization of the grape leaves and berries by *B. cinerea* using light microscopy. The following genotypes were used for each category: HR for “Ju mei gui,” and HS for “Summer black.” The leaf and berry skins were cut into 2–3 cm^2^ segments, and fixed and decolorized in 100% ethanol and in saturated chloral hydrate. The samples were stored in 50% glycerol and stained with aniline blue solution at the time of observation with an Olympus BX-51 microscope (Olympus, Tokyo, Japan) [43].

### 4.5. Scanning Electron Microscopy (SEM)

The structural growth of *B. cinerea* on leaves and berries of one representative genotype, “Ju mei gui” or “Summer black,” was observed using SEM (JEOL FESEM S-4800 scanning electron microscope, JEOL, Tokyo, Japan). Infected leaf and berry skins were cut into 1.0–1.5 cm^2^ pieces and immersed in 4% glutaraldehyde. After vacuum infiltration for 30 min, the infected leaf and berry skins were rinsed five times for 5, 10, 15, 20, 20 min, respectively, with 0.1 M sodium phosphate buffer (PBS) (pH 6.8). The segments were dehydrated in an ethanol gradient: 30%, 50%, and 70% for 15 min each; 80% and 90% for 20 min each; and 100% alcohols twice for 30 min. Finally, the samples were incubated in acetone for 30 min and isoamyl acetate twice for 15 and 30 min in three biological replicates. The segments were desiccated by CO_2_, coated with gold in a sputter coater, and then observed under a scanning electron microscope at 15 kV [43].

### 4.6. Reactive Oxygen Species

#### 4.6.1. H_2_O_2_ Determination

H_2_O_2_ levels in “Ju mei gui” and “Summer black” leaves and berries were measured at various time points (0, 8, 24, 48, 72 hpi, and 0, 2, 4, 6, 8 dpi, respectively) as previously described [44].

#### 4.6.2. O2- Determination

The “Ju mei gui” and “Summer black” leaf and berry O2- production rates were determined at time points 0, 8, 24, 48, 72 hpi, and 0, 2, 4, 6, 8 dpi, respectively, as previously described [45].

### 4.7. Antioxidant Enzymes Analyses

SOD activity was determined in extracts from “Ju mei gui” and “Summer black” leaves and berries at various time points (0, 8, 24, 48, 72 hpi, and 0, 2, 4, 6, 8 dpi, respectively) as previously described [26]. Similarly, POD activity was measured [46].

### 4.8. JA Measurements in “Ju mei gui” and “Summer black”

JA levels were measured in inoculated and control “Ju mei gui” and “Summer black” leaves and berries that were sampled at time points 0, 8, 24, 48, 72 hpi, and (0, 2, 4, 6, 8 dpi, respectively, and instantly frozen in liquid nitrogen. Further analysis was carried out as previously described [47] and JA was computed with a competitive enzyme-linked immunosorbent assay (ELISA) assay [48].

### 4.9. Statistical Analysis

For experimentation, three biological replicates were used with completely randomized design (CRD). Means and standard errors were calculated from independent replicates using SPSS 13.0. Least significant difference (LSD) 0.05 was used to calculate substantial variations in data 2019. All images were combined with the help of Adobe Photoshop. All graphs were prepared using the Origin Pro 2016 32-bit.

## 5. Conclusions

In this study, twenty-four grapevine genotypes were evaluated for leaf and berry resistance against *B. cinerea*, which revealed that one genotype was HR, one was R, eight genotypes were S, and fourteen were HS genotypes. Similarly, of the *Vitis* genotypes evaluated for grape berry: three genotypes were found to be HR, three resistant, eleven genotypes S, and seven were HS. Moreover, in the experimental results, it was indicated that the resistance of “Ju mei gui” can attribute to minor fungal growth, less ROS production, elevated antioxidant enzymes activity, and more JA contents. Furthermore, serious fungal infection of “Summer black” and persistent ROS detection coincide with rather unaffected antioxidant functions and minute JA contents. This study provides a basis for the understanding of *B. cinerea* infection and resistance mechanisms in resistant and susceptible cultivars of grapevine. 

## Figures and Tables

**Table 1:** Evaluation of disease severity in 24 *Vitis* genotypes by using leaves, post *B. cinerea* inoculation.

Species	Genotypes	a Disease Severity %	b Resistant Level	Mycelium	Sporulation	Leaf Lesion %
*V.vinifera* L.	Ju mei gui	4.6 ± 1.69	HR	N	N	8.70 ± 0.52
*V.vinifera* L.	Cinsault	92.26 ± 1.12	HS	Y	Y	87.3 ± 1.50
*V.vinifera* L.	Blue French	78.58 ± 3.93	S	Y	Y	70.00 ± 1.00
*V.vinifera* L.	Lady Finger	84.60 ± 1.03	HS	Y	Y	74.50 ± 1.32
*V.vinifera* L.	Fresno	55.75 ± 3.42	S	Y	Y	22.6 ± 2.80
*V.vinifera* L.	Merlot	97.59 ± 2.03	HS	Y	Y	86.50 ± 1.32
*V.vinifera* L.	Thompson Seedless	74.22 ± 3.66	S	Y	Y	63.33 ± 1.23
*V.vinifera* L.	Tokay	93.98 ± 1.77	HS	Y	Y	91.33 ± 1.53
*V.vinifera* L.	Flame Seedless	45.86 ± 2.48	S	Y	N	25.33 ± 1.53
*V.vinifera* L.	Zhana	63.88 ± 1.68	S	Y	Y	46.33 ± 1.53
*V.vinifera* L.	Early Muscat	96.60 ± 0.85	HS	Y	Y	94.00 ± 1.00
*V.vinifera* L.	Manaizi	92.28 ± 0.65	HS	Y	Y	92.67 ± 0.58
*V.vinifera* L.	Red Hanepoot	84.14 ± 1.68	HS	Y	Y	71.33 ± 2.08
*V.vinifera* L.	Sangiovese	76.15 ± 1.66	HS	Y	Y	62.90 ± 0.10
*V. vinifera* L. × *V. amurensis* Rupr	Bei hong	42.40 ± 2.77	S	Y	Y	22.67 ± 0.61
*V. vinifera* L. × *V. amurensis* Rupr	Beibing Hong	86.60 ± 0.43	HS	Y	Y	75.13 ± 0.81
*V. vinifera* L. × *V. labrusca* L.	Zuijin xiang	65.85 ± 1.68	S	Y	N	51.67 ± 1.15
*V. vinifera* L. × *V. labrusca* L.	Gold Finger	84.64 ± 1.56	HS	Y	Y	73.33 ± 2.89
*V. vinifera* L. × *V. labrusca* L.	Kyoho	24.77 ± 0.24	R	N	N	58.23 ± 0.68
*V. vinifera* L. × *V. labrusca* L.	Hu Tai No.8	92.67 ± 1.41	HS	Y	Y	94.27 ± 0.64
*V. vinifera* L. × *V. labrusca* L.	Tian Yuan Qi	93.07 ± 3.93	HS	Y	Y	87.83 ± 1.04
*V. vinifera* L. × *V. labrusca* L.	Summer Black	98.61 ± 1.13	HS	Y	Y	94.17 ± 0.72
*V. vinifera* L. × *V. labrusca* L.	Black Rose	76.58 ± 0.56	S	Y	Y	69.00 ± 2.00
*V. vinifera* L. × *V. labrusca* L.	Dong fang zhi xing	95.34 ± 3.16	HS	Y	Y	1.53

(a) Disease severity: The average percentage of spreading lesions determined by observation of 48 leaves. (b) Resistance level: Highly Resistant (HR: rank of 0–1.50); Resistant (R: rank of 1.51–3.50); Susceptible (S: rank of 3.51–5.50); Highly Susceptible (HS: rank of 5.51–7.0). One genotype (Table 1) was found resistant in leaves evaluation, while three genotypes (Table 2) were investigated in berries evaluation with absence of mycelium and new sporulation with SI values of 1.51–3.50.

**Table 2:** Disease severity investigation in 24 *Vitis* genotypes by using Berries with post *B. cinerea* inoculation.

Species	Genotypes	a Disease Severity%	b Resistant Level	Mycelium	Sporulation	Leaf Lesion %
*V.vinifera* L.	Ju mei gui	5.42 ± 2.42	HR	N	N	8.43 ± 0.71
*V.vinifera* L.	Cinsault	88.46 ± 1.85	HS	Y	Y	99.33 ± 1.15
*V.vinifera* L.	Blue French	73.56 ± 2.36	S	Y	Y	82.83 ± 1.76
*V.vinifera* L.	Lady Finger	55.33 ± 1.26	S	Y	Y	35.37 ± 4.45
*V.vinifera* L.	Fresno	59.19 ± 2.65	S	Y	Y	77.23 ± 1.16
*V.vinifera* L.	Merlot	76.33 ± 3.22	S	Y	Y	91.00 ± 3.00
*V.vinifera* L.	Thompson Seedless	85.33 ± 3.15	HS	Y	Y	95.00 ± 3.00
*V.vinifera* L.	Tokay	61.54 ± 3.16	S	Y	Y	68.56 ± 1.60
*V.vinifera* L.	Flame Seedless	87.11 ± 2.5	HS	Y	Y	59.56 ± 2.65
*V.vinifera* L.	Zhana	82.33 ± 3.04	HS	Y	Y	85.33 ± 1.53
*V.vinifera* L.	Early Muscat	66.48 ± 2.35	S	Y	Y	69.16 ± 0.76
*V.vinifera* L.	Manaizi	55.23 ± 0.96	S	Y	N	72.10 ± 2.17
*V.vinifera* L.	Red Hanepoot	60.52 ± 1.94	S	Y	N	88.13 ± 2.20
*V.vinifera* L.	Sangiovese	47.12 ± 1.57	S	Y	N	57.12 ± 2.19
*V. vinifera* L. × *V. amurensis* Rupr	Bei hong	18.55 ± 2.89	R	N	N	24.74 ± 1.96
*V. vinifera* L. × *V. amurensis* Rupr	Beibing Hong	4.50 ± 2.14	HR	N	N	5.50 ± 1.50
*V. vinifera* L. × *V. labrusca* L.	Zuijin xiang	24.28 ± 1.25	R	N	N	54.99 ± 4.26
*V. vinifera* L. × *V. labrusca* L.	Gold Finger	96.22 ± 0.75	HS	Y	Y	100.00 ± 0.00
*V. vinifera* L. × *V. labrusca* L.	Kyoho	19.53 ± 2.1	R	N	N	16.33 ± 1.53
*V. vinifera* L. × *V. labrusca* L.	Hu Tai No.8	77.56 ± 1.65	S	Y	Y	83.12 ± 1.88
*V. vinifera* L. × *V. labrusca* L.	Tian Yuan Qi	87.21± 2.74	HS	Y	Y	90.76 ± 1.19
*V. vinifera* L. × *V. labrusca* L.	Summer Black	98.73 ± 1.87	HS	Y	Y	91.66 ± 2.08
*V. vinifera* L. × *V. labrusca* L.	Black Rose	70.25 ± 3.14	S	Y	Y	82.50 ± 2.18
*V. vinifera* L. × *V. labrusca* L.	Dong fang zhi xing	4.83 ± 1.92	HR	N	N	1.02

(a) Disease severity: The average percentage of spreading lesions determined by observation of 105 Berries. (b) Resistance level: Highly Resistant (HR: rank of 0–1.50); Resistant (R: rank of 1.51–3.50); Susceptible (S: rank of 3.51–5.50); Highly Susceptible (HS: rank of 5.51–7.0). Mycelium and new sporulation: N= No, Y= Yes.
